# Effect of prenatal exposure to maternal cortisol and psychological distress on infant development in Bengaluru, southern India: a prospective cohort study

**DOI:** 10.1186/s12888-017-1424-x

**Published:** 2017-07-17

**Authors:** Anita Nath, Gudlavalleti Venkata Satyanarayana Murthy, Giridhara R. Babu, Gian Carlo Di Renzo

**Affiliations:** 1 Wellcome Trust DBT India Alliance Intermediate fellow in clinical and public health, Indian Institute of Public Health-Bengaluru Campus, Public Health Foundation of India, SIHFW Campus, First Cross, Magadi road, Bengaluru, Karnataka 560023 India; 2 Indian Institute of Public Health Hyderabad-Bengaluru Campus, South, Public Health Foundation of India, Plot # 1, Rd Number 44, Masthan Nagar, Kavuri Hills, Madhapur, Hyderabad, Telangana 500033 India; 3 Department of Ob/Gyn, Centre for Perinatal and Reproductive Medicine, the Midwifery School, The University of Perugia, Perugia, Italy; 4 The Permanent International and European School of Perinatal, Neonatal and Reproductive Medicine, Florence, Santa Maria della Misericordia University Hospital, 06132 San Sisto, Perugia, Italy; 5 International Center for Eye Health, Faculty of Infectious and Tropical Diseases, London School of Hygiene and Tropical Medicine, London, UK

**Keywords:** Pregnancy, Mental stress, Prenatal, Depression, Anxiety, Cortisol, Infant development

## Abstract

**Background:**

The mental health status of a pregnant woman and its consequent impact on foetal well being is not given much importance compared to the risk imposed by obstetric complications and medical conditions. Maternal psychological distress is a major public health problem and needs timely detection and intervention to prevent any adverse pregnancy outcome. There is ample evidence from literature that justifies the association of prenatal maternal mental stress and elevated cortisol with delayed infant motor and cognitive development; evidence from India being rather limited. The study aim is to prospectively assess the association of maternal psychological distress and cortisol level with motor and cognitive development of the infant.

**Methods:**

A sample of 2612 eligible pregnant women who have been registered for antenatal care at selected public sector hospitals in Bengaluru will be recruited after obtaining written informed consent. They will be assessed for the presence of maternal psychological distress in the form of depression and anxiety using appropriate scales and saliva samples will be collected for cortisol estimation during early, mid and late pregnancy. Follow up visits after delivery will be done on day 10, 3 months, 8 months and 12 months. The Bayley Scales of Infant and Toddler Development [BSID] (Third edition) will be used to measure both motor and mental milestones in terms of Psychomotor Development Index (PDI) and Mental Development Index (MDI). Logistic regression model will be used to determine the association between the exposure variables and outcomes which will be reported as Odd’s Ratio (OR) and 95% confidence intervals (CI).

**Discussion:**

Our study findings could add to the growing evidence that maternal psychological distress during pregnancy adversely influences growth and development in the offspring and subsequent development of the child. While maternal anxiety and depression can be measured by using self reporting instruments, estimation of maternal endogenous cortisol levels could serve as a biomarker of prenatal psychological stress. Findings from this study could be used to focus upon the burden of mental health problems during pregnancy and to consider steps to scale up prenatal mental health services in health care settings.

## Background

Pregnancy is a time of physiologic, mental and emotional change. As a result, women are highly vulnerable to psychological distress that includes anxiety, depression and stress [1–3]. During this phase of complex and dynamic changes, the fetal organs and organ systems that are forming are subject to both positive and negative influences, also known as foetal programming [4]. There is ample evidence from literature that justifies the association of prenatal maternal stress with delayed infant motor and cognitive development wherein it has been reported that high levels of maternal stress during mid-pregnancy was significantly associated with lower scores of motor and mental development of the infant [5–8].

Prenatal maternal stress has been frequently linked with elevated levels of maternal endogenous cortisol [9]. The cortisol hormone plays an important role in normal development of the foetus [10]. During pregnancy, the maternal cortisol levels increase by two to four times [11]. This increase has a positive influence on neural development [12]. However, foetal exposure to excess maternal cortisol may result in impaired brain development as a result of neurotoxicity [13, 14]. Animal studies show that exposure to prenatal stress induced a significant rise in maternal cortisol secretion by activating the maternal Hypothalamo Pituitary Axis (HPA). Elevated levels of cortisol reach the foetus and modify the activity of foetal HPA axis [15–17]. This modification of the foetal HPA axis exerts a detrimental effect on further growth and development through a series of complex endocrine mechanisms [18–20]. In animals, prenatal stress also exerts an inhibitory effect on the placental enzyme 11 β-hydroxysteroid-dehydrogenase type 2 which converts maternal cortisol to inactive cortisone resulting in a rise in amniotic fluid cortisol level which affects foetal HPA functioning [21].

The evidence of association of prenatal stress with elevated maternal cortisol levels in humans is rather weak [22–24]. Nevertheless, there are few studies that have evaluated the effect of prenatal exposure to elevated maternal cortisol on infant outcomes and observed an adverse effect on infant development and lower scores of IQ in childhood [5, 7, 25]. The timing of exposure to elevated maternal cortisol during the gestational period appears to be of significance. While one such study observed poor infant neuromotor development to be associated with exposure early in pregnancy, another one reported a significant association with exposure in the third trimester [5, 7].

While most of the studies have relied on maternal self reporting of psychological distress to establish its linkage with adverse infant outcome, there are a few studies in which prenatal stress did not appear to influence infant and child development [26, 27]. Thus, this kind of self reporting may have its own limitation in quantifying stress during pregnancy [5, 28]. Few of the studies have used both measures for establishing the presence of prenatal stress i.e., self reporting and measurement of maternal cortisol and observed an independent significant association with adverse infant and child outcomes in each case [5, 7, 29–31].

Evidence from India that depicts the association of prenatal stress with infant and child development is rather limited. A study done in Goa observed the existence of a positive association between psychological morbidity during pregnancy and low birth weight [32]. Another study done in India found prenatal maternal anxiety to be linked with elevated infant cortisol reactivity [33]. In another study which was undertaken to explore the relationship between prenatal stress and infant temperament, it was seen that although the levels of salivary cortisol was high in those infants whose mothers were psychologically distressed, there was no significant association with maternal reports of infant temperament [34]. However, these studies have not explored the effect of prenatal stress and maternal cortisol level on the mental and motor development of the offspring.

The prevalence of maternal prenatal depression has been observed to be a little higher than 25% in low and middle income countries [35, 36]. While core importance towards screening for obstetric risk factors and medical conditions is the norm in most settings; mental health of the pregnant women and its consequent impact on foetal well being tends to be overlooked. Maternal psychological distress is therefore a major public health problem and needs timely detection and intervention to prevent any adverse pregnancy outcome.

Given the above background, the aim of this study is to prospectively assess the association of maternal psychological distress and cortisol level with motor and cognitive development of the infant. The objectives include: (i) to describe the pattern of occurrence of maternal psychological distress during pregnancy (ii) to determine the influence of maternal psychological distress on infant motor and cognitive development (iii) to determine the influence of maternal cortisol level on infant motor and cognitive development. (iv) to correlate maternal psychological distress with maternal cortisol levels.

The proposed hypotheses are as follows:

Hypothesis 1: Maternal self reported psychological distress is associated with adverse infant motor and cognitive development.

Hypothesis 2: Elevated maternal cortisol level is associated with adverse infant motor and cognitive development.

**Fig. 1:**
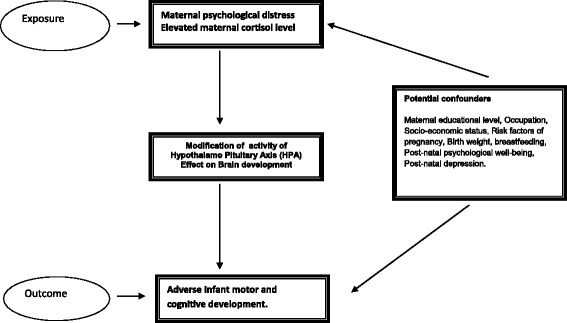
Conceptual Framework to depict Hypotheses 1 and 2

A conceptual framework of the hypothesis depicting the exposure, outcome and potential confounders is outlined in Fig. 1.

## Methods/design

### *Study design:* Prospective cohort study

#### Study participants and study setting

The study participants will include eligible pregnant women attending the antenatal clinic at the Jaya Nagar general hospital, Bangalore, who fulfill the following inclusion criteria which include (i) age between 18 and 40 years (ii) between 14 weeks to 28 weeks of gestation (iii) plan to deliver in the study hospital (iv) permanently residing in the study area within a radius of 15 km (v) full term healthy neonate with Apgar score of more than 7. Exclusion criteria will include (i) Multiple pregnancy (ii) grand multipara (iii) major pregnancy complications: preeclampsia-eclampsia, hyperemesis gravidarum (iv) birth complications: still births, preterm delivery, congenital anomalies (v) History of past or present intake of steroid medications.

#### Sample size

Evidence from literature shows that 10% to 20% of all women suffer from depression or anxiety during pregnancy [37, 38]. With an expected prevalence of 15%, an allowable error of 5%, 10% variability and power 80%, the sample size is calculated to be 2176. Assuming a drop out rate of 20%, the calculated sample size amounts to 2612.

#### Sampling method

All those pregnant women who are visiting the antenatal clinic will be screened for eligibility. The women who are found to be eligible will be enlisted every month. They will be selected to participate in the study by means of simple random sampling, by using a series of computer generated numbers. The selected women will be contacted over the phone and invited to come to the study hospital. If a selected woman denies to enter the study, the woman then with the next number will be invited to participate.

#### Study procedures

The selected women would be recruited into the study after obtaining a written informed consent from them.

The baseline assessment will be done at the time of recruitment between 14 and 28 weeks (early to mid pregnancy). A. questionnaire will be administered to collect data on socio-demographic characteristics, obstetric, medical and psychiatric past histories.

Further assessments will be done (i) 37–38 weeks of gestation (late pregnancy), and at (ii) 10 days (iii) 3, 8 months (iv) 12 months after birth.

**Fig. 2:**
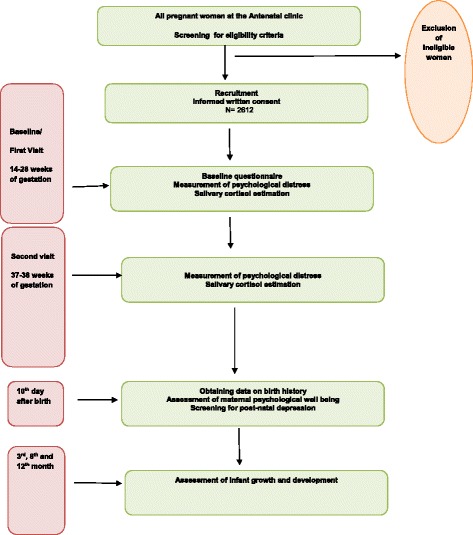
Flowchart depicting steps of the cohort study

A flowchart depicting the study steps is shown in Fig. 2.

### Measurement of study exposure and outcomes

#### Maternal psychological distress

The presence of maternal psychological distress will be identified by using Kessler 10 Scale of Psychological Distress (K10) for prenatal depression [39] and the 10-item Pregnancy related anxiety (PRA) scale for pregnancy anxiety [40]. The K10 scale is well structured and includes questions about anxiety and depressive symptoms that a person has experienced in the most recent 4 week period. Each question has five response categories ranked on a five-point scale, with the score being the sum of these responses. The PRA scale measures concerns and worries that are specific to pregnancy and childbirth. The scoring on this scale ranges from 10 to 40. These assessments will be done at (i) Baseline visit - between 14 and 28 weeks (ii) Second visit: 37–38 weeks.

The levels of the unbound cortisol hormone in blood is reliably reflected by salivary cortisol levels [41]. Maternal saliva samples will be collected for cortical analyses at (i) Baseline visit - between 14 and 28 weeks (ii) Second visit: 37–38 weeks.

The salivary samples will be stored until assayed at −70 °C, in a Biorepository facility at the St. Johns Research Institute. The evaluation of the samples will be done at an NABL (National Accreditation Board for Testing and Calibration Laboratories) accredited Lab. Before assay, the samples are thawed and centrifuged for about 15 min. The cortisol levels are estimated by a competitive luminescence immunoassay with a lower limit of detection of 0.5 nmol/L [42]. The infant will be assessed at 3, 8 and 12 months for developmental indices by the Bayley Scales of Infant and Toddler Development (BSID), Third edition which measures both motor and mental milestones [43]. This will be accomplished by means of home visits. The Bayley scales cover five major areas of development - cognitive, communication, physical, socio-emotional and adaptive. The scales to measure the cognitive and physical areas of development will be used. The weight and length of the infant will also be recorded during these visits.

All the psychometric measurement scales and BSID will be translated into the local language *‘Kannada’* using the standardised WHO translation and back-translation protocol (WHO) and pre-tested before being finalized [44].

The Research team will receive training for using these scales from a team of experts at CSI Holdsworth Memorial Hospital, Mysore.

### Confounders

The confounding factors that influence the outcome variables- cortisol level and infant growth and development will be taken into account at the time of data analysis. These are enlisted in Fig. 1. Data on maternal educational level, occupation, socio-economic status, pregnancy risk factors will be obtained from the baseline information. The gestational age will be calculated from the last menstrual period as reported by the study participants. Information on birth weight will be obtained from medical records. Information on breastfeeding, post-natal psychological well-being and post-natal depression will be collected at the 10th day visit after birth. Maternal psychological well being will be assessed by using the General Health Questionaiire-30 which measures one’s ability for effective daily functioning [45]. This contains 30 questions which are answered on a 4- point scale. Post natal depression will be measured by using the 10-item Edinburgh Postnatal Depression Scale (EPDS) [46].

### Data management and analysis

Data management will be done using Microsoft Access which will be transported to SPSS Version 24 for analysis. Descriptive statistics will be used to summarize the socio-demographic characteristics, obstetric variables, perinatal outcomes, scores obtained from measurement scales and cortisol level estimates. Confounding variables will be tested for their relationships with the exposure (Maternal anxiety, depression and salivary cortisol level) and outcome (Mental Development Index and Psychomotor Development Index) by means of correlations (Pearson correlation coefficient or Spearman rank-order correlations where appropriate) and regression analysis. Only those variables that are significantly related to the exposure and outcome will be included in further analyses. Logistic Regression Model will be used to determine the association between the exposure variables and outcome which will be reported as Odd’s Ratio (OR) and 95% confidence intervals (CI). The statistical significance for all the tests will be assumed to be at the level of *P* < .05.

### Ethical considerations

The protocol for the proposed study has been reviewed and approved by the institutional ethical review board (IEC) at Bengaluru campus of IIPH-H. All the study participants will be required to provide a written informed consent in the local language.

## Discussion

Our study findings could add to the growing evidence that maternal psychological distress during pregnancy adversely influences growth and development in the offspring and subsequent development of the child. While maternal anxiety and depression can be measured by using self reporting instruments, estimation of maternal endogenous cortisol levels could serve as a biomarker of prenatal psychological stress. Perhaps a combination of the above mentioned subjective and objective measures could give a better measure of mental stress during the antenatal period.

The findings from this study could be used to focus upon the burden of mental health problems during pregnancy with its adverse consequences and to consider steps to scale up prenatal mental health services in both public as well as private health settings. The study findings could be used to plan for evidence based- interventions such as stress reduction programmes to reduce maternal anxiety and depression during pregnancy, particularly in the Indian scenario.
